# Evolutionary dynamics of selfish DNA explains the abundance distribution of genomic subsequences

**DOI:** 10.1038/srep30851

**Published:** 2016-08-04

**Authors:** Michael Sheinman, Anna Ramisch, Florian Massip, Peter F. Arndt

**Affiliations:** 1 Max Planck Institute for Molecular Genetics, Berlin, Germany; 2 Department of Biology, Faculty of Science, Utrecht University, Utrecht, the Netherlands; 3 INRA, UR1404 Mathématique Informatique Appliquées du Génome á l’Environnement-F-78350 Jouy-en Josas, France

## Abstract

Since the sequencing of large genomes, many statistical features of their sequences have been found. One intriguing feature is that certain subsequences are much more abundant than others. In fact, abundances of subsequences of a given length are distributed with a scale-free power-law tail, resembling properties of human texts, such as Zipf’s law. Despite recent efforts, the understanding of this phenomenon is still lacking. Here we find that selfish DNA elements, such as those belonging to the Alu family of repeats, dominate the power-law tail. Interestingly, for the Alu elements the power-law exponent increases with the length of the considered subsequences. Motivated by these observations, we develop a model of selfish DNA expansion. The predictions of this model qualitatively and quantitatively agree with the empirical observations. This allows us to estimate parameters for the process of selfish DNA spreading in a genome during its evolution. The obtained results shed light on how evolution of selfish DNA elements shapes non-trivial statistical properties of genomes.

Our genome is a sequence of A, C, G and T nucleotides and can be viewed as a long text of about three billion letters. Only a small part of our genome is functional and under selection^1^,^2^,^3^; the rest (so-called junk DNA) mostly evolves neutrally and, therefore, is naively expected to be a random sequence. However, the junk DNA contains many homologous sequences, sharing significant similarities to each other. Hence, its statistical properties differ from those of random sequences^4^,^5^,^6^,^7^. One of these properties, which we discuss here, is that for a given length, certain subsequences are much more abundant than others^8^,^9^,^10^,^11^. Namely, the abundances of *k*-mers—sequences of length *k*—possess a wide, scale-free distribution, as shown in Fig. 1. One can see that even for large values of *k*, one finds *k*-mers which appear more than 10^4^ times in the human genome, while in a randomly shuffled genome such *k*-mers would be unique.

This phenomenon resembles statistical properties of human texts, where abundances of words also exhibit a scale-free distribution^12^. For human texts such a linguistic feature is often presented as Zipf’s^13^,^14^ or Heaps’^15^ law. We exemplify the similarity between the statistics of *k*-mers in the human genome and the statistics of words in human texts in Fig. 1. Despite an incomplete analogy, caused by the lack of a natural definition of a word in the genomic context, this intriguing similarity between the genome and human texts has led some researchers to analyze genetic sequences from a linguistic perspective (see, e.g., refs 16,17). However, beyond human texts, many types of data in the physical and social sciences can be approximated with a Zipf distribution, so that the observation that genomic texts obey Zipf’s law is not enough to prove genomes have linguistic features. Linguistic features include morphology, syntax, and semantics and Zipf’s distribution alone is not specific to language. This is why several studies called linguistic properties of genomes into question^18^,^19^,^20^,^21^,^22^,^23^,^24^,^25^. Here we present an explanation for the observed genomic phenomenon, showing that the *pseudo*-linguistic features of the *k*-mer abundances statistics in genomes are a consequence of selfish DNA expansion in our genome during its evolution. We develop a model, which accurately reproduces statistical properties of abundant subsequences in the genome. The model is based solely on selfish spreading of DNA repeats, demonstrating that high abundances of certain *k*-mers do not reflect their functionality for the organism.

Considering different compartments of the genome, one finds that the scale-free distribution of abundances is dominated by subsequences of selfish repetitive DNA elements (see Fig. 1). This suggests that the scale-free distribution of abundances is a consequence of the evolutionary dynamics of such elements. Selfish DNA elements (or repeats) are parasitic sequences that duplicate with the help of the cellular machinery of the host organism^26^,^27^. Such duplications significantly increase the size of genomes during their evolution and often appear in bursts of activity during a few tens of millions of years^28^,^29^. After such a burst, the duplication activity stops, but the existing repetitive elements remain in the genome. Some elements acquire a function^30^ or cause a disease^31^, but most fade away neutrally into the genomic background due to mutations^32^. One of the largest and most studied families of repeats in primates is the Alu family, covering 15% of the human genome with more than a million copies^33^,^34^. In the following, we use the Alu family as a model system to study statistical properties of selfish DNA sequences.

To gain insight into the origin of the observed fat-tailed distributions of *k*-mer abundances in the human genome, we plot in Fig. 2 the distributions for the Alu family elements for different values of *k* (see Methods for details). One can see that even the abundances of short *k*-mers are much more dispersed than in a random sequence. For large values of *k* > 20 the distributions possess a power-law tail, i.e. *n*_*k*_(*s*) ~ *s*^−*α*^. Importantly, the exponent of the power-law distribution, *α*(*k*), depends on *k*, such that it increases with *k*, starting from about 2 for small values of *k*. This dependence is clearly visible in Fig. 3, where we measure the values of *α*(*k*) using the Hill estimator (see Methods). A model for the evolutionary dynamics of selfish DNA ought to be able, in particular, to explain these properties of the power-law exponent and, in general, to reproduce the empirical distributions.

In this paper we present a simple model for the evolution of selfish DNA, which accounts qualitatively and quantitatively for the observed distributions of *k*-mer abundances. Using our model, we estimate key parameters of the spreading dynamics of Alu repeat elements and compare them to previous estimates. Our results demonstrate that some non-trivial properties of genomic texts can be understood considering the evolution of selfish DNA, without referring to any linguistic structure of genomes.

## The model

We analyze a model for the evolution of selfish DNA in a genome, which is based on the assumption that every repeat element can be active and duplicate with a certain rate or passive and does not duplicate at all. In a sense our model is a mixture between the transposon model, which assumes that every Alu element duplicates with the same rate^35^ and the master gene model, which assumes that there is a single active, duplication potent element which gives rise to all other silent elements^36^,^37^. Our approach of mixing the two models is supported by recent studies, which suggest that the fraction of active Alu elements is not 100% as in the transposon model nor extremely small as in the master gene model^38^,^39^,^40^.

In our model the process starts from the appearance of a single active (i.e. able to duplicate) selfish element of length *L* at time *t* = 0. During the burst of activity, in the time interval 0 ≤ *t* ≤ *T*_1_, all existing *active* elements duplicate in a genome with rate *γ*. Each duplication results in a new identical element, which we assume to be *active* with probability *δ* and silent (non-duplicating) with probability 1 − *δ*. This results in an exponential growth, such that the average number of elements after the burst of activity ends at time *t* = *T*_1_ is given by


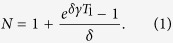


During such a burst, these duplications shape a branching process, which gives rise to a phylogenetic tree, as illustrated in Fig. 4. After the burst, the duplication activity is suppressed and all *N* elements are silenced for a time period *T*_2_. We observe these elements in the present day genome, at time *t* = *T*_1_ + *T*_2_. During and after the burst, all existing elements accumulate mutations with rate *μ*_0_ per bp, except CpG nucleotides, which mutate approximately 6 times faster^41^. We define the effective mutation rate *μ* as the weighted average of the two rates. An illustration of the described model for the evolution of selfish DNA elements is presented in Fig. 4.

To address the empirically observed scale-free distribution of *k*-mer abundances in genomic data, we consider in particular the statistics of *k*-mers in this model. There are *L* − *k* + 1 *k*-mers in a single element. A duplication event increases the number of all *k*-mers in the duplicating element, while mutations decrease abundances of certain *k*-mers and increase abundances of others. The mutation rate of a *k*-mer is *μk*, such that the probability that a *k*-mer does not mutate for a time *T*_2_ is given by 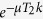.

Using two simplifying assumptions, we solve the model analytically (see detailed derivation in the SI). The analytic solution of the model yields that the number of *k*-mers with abundance 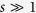 at present time *t* = *T*_1_ + *T*_2_, which we denote by *n*_*k*_(*s*), is given by


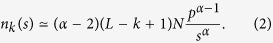


Here *N* is the number of repeat elements at present time, given by Eq. (1). The power-law exponent of the distribution is


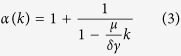


and 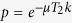 is the probability of a *k*-mer to preserve its sequence during the second, silent phase.

Note, that the power-law tail exists only if, on average, a *k*-mer duplicates faster than it mutates, such that *μk* < *δγ*. In the context of this paper this condition is fulfilled. The derived dependence for the power-law exponent *α* in Eq. (3) accounts for the observations presented above: *α*(*k*) is predicted to increase with *k*, starting from *α*(*k*) = 2 for small values of *k*.

To further quantitatively test the presented model, one needs to estimate the parameters *N* (number of elements), *μ* (effective mutation rate), *T*_1_ (time of the first, active phase), *T*_2_ (time of the second, silent phase) and *δ* (probability of a new element to be active). The duplication rate, *γ* can be then estimated using Eq. (1). We obtain the estimates using the empirical data and the analytic result (3). As we show below, our model accurately reproduces the empirical data for the Alu family of repeats for the estimated set of parameters.

## Modeling evolution of Alu repeats

The presented model can be used to study the evolution of large selfish DNA families. We apply it here to the Alu family of repeats, studying distributions of *k*-mer abundances in all identified Alu repeats in the human genome, excluding the still active AluY subfamily^42^ (see Methods for more details). In Fig. 2 one can see that these distributions qualitatively agree with the predictions of Eqs (2) and (3): the tails of the distributions follow a power-law, the exponents of these power-laws are larger than 2 and grow with *k* (see also Fig. 3).

We start now with the estimation of the parameters of the model. First, we estimate the ratio 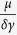 using the analytic result (3). From the empirical data the value of the power-law exponent *α*(*k*) can be estimated for each *k* using the Hill maximum-likelihood estimator^43^ (see Methods). In Fig. 3 one can see the agreement of Eq. (3) with the empirical power-law exponents, using a fit with a single free parameter, 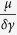. The fitting results in


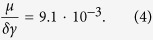


To estimate the effective mutation rate, *μ*, it is important to consider hypermutable CpG di-nucleotides along the Alu elements. In fact, *μ* is the average mutation rate of CpG di-nucleotides and other nucleotides. There are 24 CpG di-nucleotides in a typical Alu element (e.g. in the consensus sequence of the AluSx subfamily) of length *L* ≃ 300. These di-nucleotides mutate about 6 times faster than other nucleotides on both positions^41^. Thus,


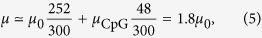


where *μ*_0_ is the mutation rate of the non-CpG nucleotides and *μ*_CpG_ = 6*μ*_0_ is the mutation rate of the CpG nucleotides. In the following we measure all the rates in units of *μ*_0_, which is of the order of 10^−9^ yr^−1^, and times in units of 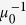. The value of *μ*_0_ is just a global time scale and does not affect the *k*-mer abundances. Nevertheless, in the Discussion section we estimate *μ*_0_, convert all estimated parameters to standard units and compare them with previous estimates in the literature.

The probability of a new element to be active, *δ*, does not affect the results in the asymptotic limit of large numbers of Alu elements, *N*, as long as the effective duplication rate, *δγ*, is kept constant. However, for a finite value of *N* the results change if *δ* is too small. In this case estimates of *α*(*k*) would be biased to higher values due to highly abundant copies of several active elements, such that Eq. (3) would not fit well the biased estimates. The fact that Eq. (3) does fit well the empirical data indicates that *δ* is not very small. Our simulations, with *N* = 776710 and Eq. (4), indicate that the distribution of abundances does not depend significantly on *δ* and Eq. (3) fits well the data, as long as *δ* is above 5%. This result supports an earlier study, where *δ* is estimated to be 10–20%^44^. Using our estimate *δ* = (5–100)% and Eqs (4) and (5) we conclude that the duplication rate is in the range *γ* = (0.2–4) ⋅ 10^3^*μ*_0_. Furthermore, using the above estimates together with Eq. (1) we get the estimate *T*_1_ = (5.3–6.9) ⋅ 10^−2^/*μ*_0_.

To find the only remaining missing parameter *T*_2_, we use the independence of the results on the value of *δ* in the relevant regime, setting *δ* = 1 and simulating the model for many different values of *T*_2_. The best agreement between the empirical distribution of abundances and the simulated one was obtained for


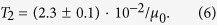


More details about the estimation of the parameters from the empirical data can be found in Methods.

In summary, the estimated parameter set for the Alu family evolution model is


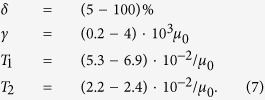


As shown in Fig. 2, the model with this set of parameters accurately reproduces the empirical distributions of the *k*-mer abundances for the Alu elements.

## Discussion

There are a few important things to note before we draw conclusions and summarize. First, the presented model is similar in spirit to the one suggested in ref. 10. However, the basic assumption there was that the evolution of the selfish DNA elements approaches a steady state with a constant genome size, such that any new element replaces an old one, resulting in 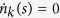. As has been shown in ref. 10, this assumption can only result in an abundance distribution following 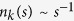, such that *α* = 1 for all values of *k*. In contrast, we assume an exponentially growing steady state of the genome in the burst phase, 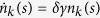 (see SI). The last assumption makes more biological sense for the expansion of selfish DNA, with a weak or no selection against it. Only in this case, when there is a phase of exponentially expanding repeats, one can get a power-law exponent *α*(*k*) which is always larger than 2 and depends on *k*, as it is observed for the empirical data.

In our model we assumed that CpG di-nucleotides mutate 6 times faster than other nucleotides. This assumption results in an effective mutation rate of Alu elements which is 1.8 times higher than the mutation rate of non-CpG nucleotides elsewhere in the genome (see Eq. (5)). A tempting simplification of the model would be to ignore the CpG di-nucleotides, assuming an effective mutation rate for all nucleotides along an Alu element. However, in that case, the distribution obtained for *k*-mer abundances is qualitatively different from the empirical distribution (see SI for more details). Here, we only stress that modeling non-uniform mutation rate with highly mutable CpG di-nucleotides is essential to account for the empirical data.

Evolution of repeat elements in our genome is a complex process, which probably involves selection, population dynamics and other factors^28^,^45^,^46^,^47^,^48^. Detailed studies of Alu repeats reveal a complex history with many subfamilies appearing at different times^29^,^49^,^50^,^51^,^52^,^53^,^54^. As it often happens in nature, very complex phenomena tend to exhibit random-like statistical features. For instance, complex speciation processes result in a simple Yule statistics of genera sizes^55^ and simple statistics of pairwise genomic distances^56^; complex biochemical processes result, to some extent, in simple molecular clocks with effective mutation rates of nucleotides and amino acids on the evolutionary timescale^57^,^58^, etc. This study suggests another example of this kind: a complex evolution of selfish DNA elements exhibits random-like properties with some effective parameters.

Our estimates of those effective parameters might suffer from various biases. The first stems from the fact that we assumed a constant mutation rate along the human lineage since the origin of the Alu family in the genome, which might have varied, for instance due to different generation times^59^,^60^. Moreover, Alu elements are enriched (relative to the whole genome) with CpG di-nucleotides which possess an order of magnitude higher mutation rate^41^,^61^,^62^. We assumed that the mutation rate of the CpG di-nucleotides is 6 times higher than that of other nucleotides, but in reality the situation might be more complex^41^. Positive or negative selection can increase or decrease the estimate for the effective mutation rate. The duplication rate can possess more complex temporal structure than the one we assumed in our model and may depend on the sequence of the element^29^. These and other effects are, probably, the reason for the disagreement between our simulations and the empirical distributions at small abundances and, for very long *k*-mers, at the very end of their tails (see Fig. 2). Another possible reason for this disagreement is an intrinsic stochasticity of the simulations. Performing other simulations with a different random seed, one finds that the simulations disagree in the same regions where the simulation disagrees with the empirical results (see Fig. 2 and Fig. S2 in SI). This means that in those regions using our stochastic model one cannot fit the empirical results because the results of the simulations significantly depend upon the realization of the model (random seed). In other regions, however, the results of the simulations are very robust to the stochastic noise and agree well with the empirical results.

Our estimate for the age of the Alu family in units of the neutral mutation rate is *T*_1_ + *T*_2_ = (7.7–9.3) ⋅ 10^−2^/*μ*_0_. Alternatively, one can estimate the age of the Alu family from the following phylogenetic arguments. The Alu family is primate specific^63^, so we expect that the age of Alu is about 80 ⋅ 10^6^ yr^64^. Therefore, our estimate for the neutral mutation rate turns out to be about *μ*_0_ = (0.9–1.1) ⋅ 10^−9^ yr^−1^, within the range estimated in the literature^60^. Furthermore, in this case our estimate of *T*_2_ = (20–24) ⋅ 10^6^ yr is in a rough agreement with the conclusion of ref. 65 that “most human Alu sequences were inserted in a process that ceased about 30 million years ago”. A similar estimate was obtained in ref. 36. Therefore, our estimates of the parameters yield reasonable values, suggesting that the above discussed possible biases are not of great importance in the context of this study.

In the literature there are two alternative models for the expansion of Alu elements. The first one is the transposon model, which assumes that every Alu element duplicates with the same rate^35^. This corresponds to *δ* = 1 in our model. The second one, the master gene model, assumes *δ* = 0, implying that there is a single active, duplication potent element which gives rise to all other elements^36^,^37^. More recent studies suggest that the fraction of active Alu elements, *δ*, is not 100% as in the transposon model nor extremely small as in the master gene model^38^,^39^,^40^. This fraction for the Alu family was estimated to be 10–20%^44^. From our simulations we find that Eq. (3) is expected to fit the data well as long as *δ* is larger than 5%. Therefore, the fact that the empirical data is well fitted by Eq. (3) (see Fig. 3) supports the estimate in ref. 44.

Since the assumptions of our model are quite general, it is expected to capture the dynamics of evolution of many selfish elements in many species. Indeed, abundance distributions of *k*-mers of the Alu elements in the chimp genome are very similar to the ones in the human genome (see Fig. S3 in SI). Beyond the Alu family, several large repeat families in other species distant from human demonstrate the behaviour predicted by our model: the *k*-mer abundance distributions possess a power-law tail, its power-law exponent is above 2 and grows with *k*, following Eq. (3) (see Figs S4–S7 in SI).

Selfish elements cover a significant fraction of our genome, resulting in a genome-wide power-law distribution of *k*-mer abundances. Since different selfish DNA families evolve with different effective parameters, their mixture—the genome-wide power-law—is not expected to be clean. However, the main predicted feature of our model is that the power-law exponent has to be larger than and close to 2, as it is observed. Thus, our results explain the genome-wide power-law in th *k*-mer abundance distributions and, therefore, in the Zipf plot of *k*-mers in genomes, without referring to any linguistic or functional features. In fact, we demonstrate that a high abundance of a certain subsequence is not necessarily due to its functionality for an organism, but may rather reflect its ability to parasitize and selfishly spread in the host genome. The presented simple model of selfish DNA evolution surprisingly accurately accounts for statistical properties of these highly abundant subsequences in our genome.

## Methods

### Data

**Figure 1:**
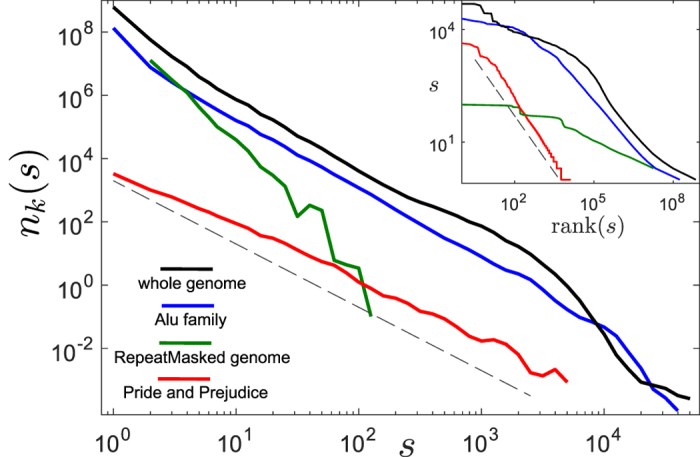
Distributions of abundances of *k*-mers for *k* = 40. *s* is the number of copies of a certain *k*-mer and *n*_*k*_(*s*) is the number of different *k*-mers with abundance *s*. Distributions for different genomic compartments are presented: the whole genome (solid, black), the whole genome after masking the repeat elements (solid, green) and the Alu family of repeats (solid, blue). For comparison the distribution of word abundances in Pride and Prejudice^72^ is also shown (solid, red). The dashed line represents the power-law *n*_*k*_(*s*) ~ *s*^−*α*^ with *α* = 2. For a randomly shuffled human genome or a random sequence of the same length there is not a single *k*-mer with *s* > 1. Inset: the corresponding Zipf’s plots for the main figure. For each *k*-mer (or a word for Pride and Prejudice) its abundance is plotted vs. the rank of its abundance. The dashed line represents the power-law *s* ~ 1/rank(*s*).

**Figure 2:**
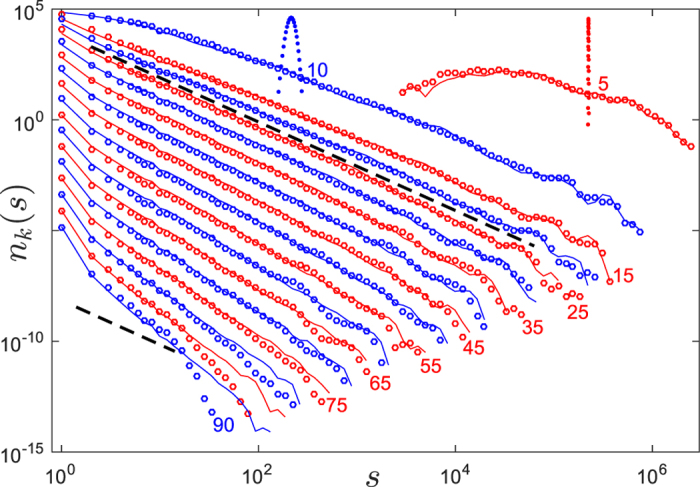
Distributions of abundances of *k*-mers, *n*_*k*_(*s*), for different values of *k*, from 5 to 90 in steps of 5, from top to bottom (see numbers in the figure). Circles represent *n*_*k*_(*s*) in the empirical data for the Alu family of repeats (see Methods). Dots represent *n*_*k*_(*s*) in a random sequence, of the same length as the empirical one for *k* = 5 (red) and *k* = 10 (blue). Lines represent *n*_*k*_(*s*) in simulated Alu elements using the set of parameters in Eq. (9) in Methods. The results of the simulations do not change drastically, as long as the parameters are within the ranges specified in Eq. (7). The dashed lines represent the power-law decay *n*_*s*_ ~ *s*^−*α*^ with *α* = 2. For visibility the values of *n*_*k*_(*s*) are normalized differently for each value of *k* (but in the same way for the empirical and the simulated data), so that the units of the vertical axis are arbitrary.

The sequences of all identified Alu repeat elements in the human genome were downloaded from the Ensembl database using the Perl API^66^. We filter out the X and Y chromosomes and the sequences belonging to the still active AluY subfamily^42^. In Fig. 1 the AluY subfamily is not filtered out. Sequences of other repeat elements in other species were downloaded using the table browser from the UCSC Table Browser data retrieval tool^67^. The *k*-mer abundances were counted using the Jellyfish program^68^. To smooth the resulting distribution of abundances in Fig. 2, we used logarithmic binning for *s* > 7, such that the ratio between two neighbouring values of *s* is 1.1885.

### Simulations

First we computed a branching pattern (or phylogenetic tree) of selfish elements as shown schematically in Fig. 4. This branching process was simulated in continuous time using a Kinetic Monte Carlo scheme^69^. We start with one active element at *t* = 0. At any given point in time all possible future branchings of active elements are listed; each of them occur with rate *γ*. Drawing a random number one of them is selected and executed; the time then advanced appropriately. The new element is active, i.e. able to duplicate again, with probability *δ*. Drawing another random number we determine whether the new edge is active or not. When the total number of elements approaches the empirical one *N* = 776710 we rescale the length of all edges, such that the height of the tree is *T*_1_. After this the terminal edges of the tree are extended by the time *T*_2_, such that the height of the tree is *T*_1_ + *T*_2_.

Once the phylogenetic tree is computed, we simulate the evolution of nucleotide sequences along its edges. At the root we start with the ancestral AluSx master sequence, which is mutated along the edges and duplicated at the nodes of the phylogenetic tree. Mutations are again modeled by Kinetic Monte Carlo. Nucleotides change to one of the other 3 nucleotides with rate *μ*_0_. To model the hyper-mutability of CpGs, we allow Cs and Gs in CpGs to change to T or A, respectively, with an increased rate *μ*_C*pG*_ = 6*μ*_0_.

### Estimating parameters and fitting procedures

The number of Alu elements in the empirical data we estimate as the average of 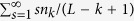 over all values of *k* in the range 5 ≤ *k* ≤ 90, with *L* = 300. This results in *N* = 776710.

Using the empirical data we estimate the value of the power-law exponent of the *k*-mer abundances distributions tail, *α*(*k*), using the Hill maximum-likelihood discrete estimator^43^,^70^ for *s* ≥ 3. Namely, for each *k*,


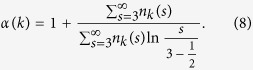


**Figure 3:**
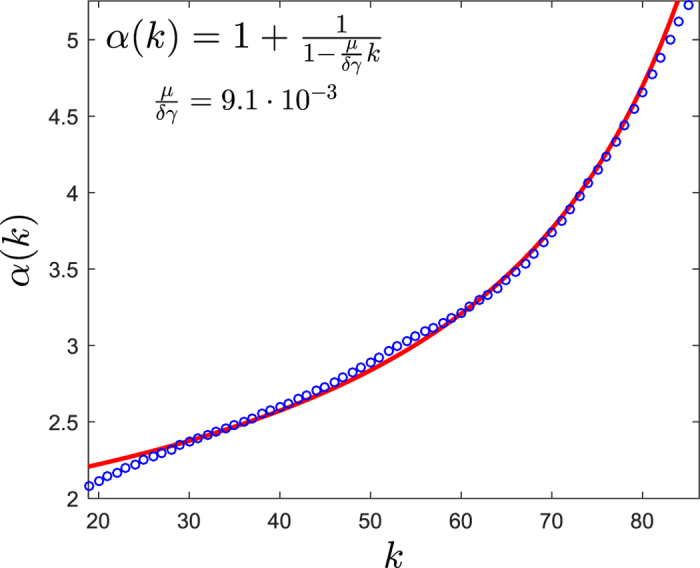
Estimation of parameters of the model using the analytic fit of the empirical data. Circles represent the empirical power-law exponent *α* as a function of *k*. The line is the numerical fit of the data points using Eq. (3), resulting in Eq. (4). The analytic equation for the fit and the resulting estimator are presented in the upper-left corner. For details of the estimators and the fit see Methods.

**Figure 4:**
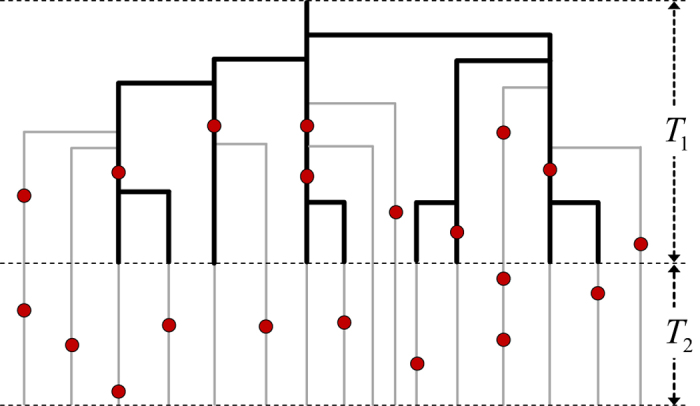
Illustration of the analysed model for the dynamics of repeat elements. Each branch represents a sequence of the repeat. Active elements are depicted in thick, black lines, while silent ones are shown in thin, gray lines. During the activity burst, selfish elements duplicate exponentially with time and accumulate mutations (red marks). After the burst sequences do not duplicate anymore but still mutate.

The obtained values of *α*(*k*) are fitted in the range 35 ≤ *k* ≤ 75 with Eq. (3) with 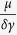 as a single free parameter [see Fig. 3] using the Levenberg-Marquardt nonlinear least squares algorithm^71^ in Matlab. This way we get the estimate (4).

The value of *T*_2_ we fit manually by simulating the model with many different values of *T*_2_ with *δ* = 1. This results in estimate (6). With estimates (4) and (6) we simulate the model changing the value of *δ*. The results do not change significantly from the *δ* = 1 case, as long as *δ* is not below 5%. Furthermore, below this threshold one cannot fit the empirical estimates of *α*(*k*) with Eq. (3). Since the empirical results are well fitted with Eq. (3), we conclude that *δ* is the range 0.05 ≤ *δ* ≤ 1. The resulting set of estimated parameters used for simulations is


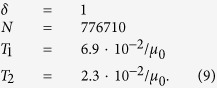


The results of the simulations of the model with these parameters can be seen in Fig. 2. For *δ* in the estimated range 0.05 ≤ *δ* ≤ 1 the estimates for the parameters of the model are given in Eq. (7).

## Additional Information

**How to cite this article**: Sheinman, M. *et al*. Evolutionary dynamics of selfish DNA explains the abundance distribution of genomic subsequences. *Sci. Rep.* **6**, 30851; doi: 10.1038/srep30851 (2016).

## Supplementary Material

Supplementary Information
