# Supplementary material for: Evolutionary dynamics of selfish DNA explains the abundance distribution of genomic subsequences
Source: Sci Rep. 2016 Aug 4;6:30851. doi: 10.1038/srep30851 (PMC4973250; doi:10.1038/srep30851)
Supplement: Supplementary Information [file srep30851-s1.pdf]

Evolutionary dynamics of selfish DNA explains the abundance distribution of genomic subsequences

Michael Sheinman[1,2,*], Anna Ramisch[1], Florian Massip[1,3], and Peter F. Arndt[1]

[1]Max Planck Institute for Molecular Genetics, Berlin, Germany
[2]Department of Biology, Faculty of Science, Utrecht University, Utrecht, the Netherlands
[3]INRA, UR1404 Mathématique Informatique Appliquées du Génome à l'Environnement-F-78350 Jouy-en Josas, France
[*]mishashe@gmail.com

ABSTRACT

Since the sequencing of large genomes, many statistical features of their sequences have been found. One intriguing feature is that certain subsequences are much more abundant than others. In fact, abundances of subsequences of a given length are distributed with a scale-free power-law tail, resembling properties of human texts, such as the Zipf's law. Despite recent efforts, the understanding of this phenomenon is still lacking. Here we find that selfish DNA elements, such as those belonging to the Alu family of repeats, dominate the power-law tail. Interestingly, for the Alu elements the power-law exponent increases with the length of the considered subsequences. Motivated by these observations, we develop a model of selfish DNA expansion. The predictions of this model qualitatively and quantitatively agree with the empirical observations. This allows us to estimate parameters for the process of selfish DNA spreading in a genome during its evolution. The obtained results shed light on how evolution of selfish DNA elements shapes non-trivial statistical properties of genomes.

Supplementary information (SI)

Analytic model and its solution

To solve the model of selfish DNA evolution analytically we consider two simplifying assumptions:

1. If a mutation happens in a k-mer this k-mer becomes a new, unique sequence in the genome. While this assumption is valid for large values of k, the mutated k-mer has a significant chance not to be unique, i.e., to be present elsewhere in the genome, for small values of k. That is why the abundance distribution of short k-mers is not well described by the analytic model, but agrees with the results of the simulations of the full model without the simplifying assumptions. And this is the reason why we fit the parameters using the analytic solution only for large values of $k \geq 30$.

2. The second assumption is that all elements are active but with the reduced effective duplication rate $\delta\gamma$.

Within this framework, let us consider now $n_k(s,t)$—the average number of different k-mers, which appear s times in all copies of the repeat during the burst, at time $0 \leq t \leq T_1$. Starting from a single element the initial condition is given by $n_k(s,t=0) = (L-k+1)\delta_{s,1}$. The dynamic equation for $n_k(s,t)$ for $s>1$ is given by

$$\dot{n}_k(s,t) = \delta\gamma(s-1)n_k(s-1,t) + +\mu k(s+1)n_k(s+1,t) - s(\delta\gamma + \mu k)n_k(s,t). \quad \text{(S1)}$$

The first term is the gain of $n_k(s,t)$ from duplications of k-mers which appear $s-1$ times. The second is the gain term from mutations (the effective mutation rate for a k-mer is μk, assuming independently mutating base-pairs) of k-mers which appear $s+1$ times. The third term is the loss of $n_k(s,t)$ from duplications or mutations of k-mers which appear s times. The dot denotes the time derivative.

Every mutation of a k-mer is assumed to generate a unique k-mer, with abundance $s=1$. This is reflected in the equation for $n_k(s=1,t)$, which takes the form

$$\dot{n}_k(1,t) = \mu k\left[N(t)(L-k+1) - n_k(1,t) + 2n_k(2,t)\right] - \delta\gamma n_k(1,t), \quad \text{(S2)}$$

where

$$N(t) = 1 + \frac{e^{\delta\gamma t} - 1}{\delta} \quad \text{(S3)}$$

is the total number of repeat elements at time t. The gain term in Eq. (S2) is due to mutations of all repeat elements (excluding those with abundance $s=1$). Note that a mutation of a k-mer with $s=2$ generates two k-mers with $s=1$. The loss term is due to duplications of k-mers with $s=1$ copies.

The equations for the dynamics of the abundances distribution during the burst phase, (S1) and (S2), can be solved analytically to any required precision in the steady state limit, which in this burst phase exhibits an exponential growth of $n_k(s,t)$ with rate $\delta\gamma$, for all finite s values, such that

$$\dot{n}_k(s,t) = \delta\gamma n_k(s,t). \tag{S4}$$

In this limit the solution of Eq. (S1) for large values of s is given by

$$n_k(s,t) \simeq (\alpha-2)(L-k+1)N(t)\frac{1}{s^{\alpha}}, \tag{S5}$$

where the power-law exponent α is given by Eq. (3). The prefactor is obtained using the normalization condition,

$$\sum_{s=1}^{\infty} s n_k(s,t) = (L-k+1)N(t). \tag{S6}$$

After the burst ends at time $t=T_1$, abundances of non-unique k-mers decrease on average due to mutations. The probability of a k-mer to preserve its sequence for time T_2 without mutations is $p=e^{-\mu T_2 k}$. Thus, the distribution of abundances for $s>1$ at present time $t=T_1+T_2$ is given by

$$n_k(s,t=T_1+T_2) = \sum_{j=s}^{\infty} n_k(j,T_1)\binom{j}{s} p^s(1-p)^{j-s}. \tag{S7}$$

For $s=1$ the number of k-mers is further increased after the burst due to mutations of non-unique k-mers and is given by

$$n_k(1,t=T_1+T_2) = \sum_{j=1}^{\infty} n_k(j,T_1)\binom{j}{1} p^1(1-p)^{j-1} + (L-k+1)N(1-p). \tag{S8}$$

Saddle point approximation of Eq (S7) (the saddle point is at $j=s/p\gg 1$) results in Eq. (2).

Importance of CpG di-nucleotides

To assess the importance of the non-uniform mutation rate with 6 times more mutable CpG di-nucleotides we performed simulations with a uniform mutation rate equal to the effective one, $1.8\mu_0$ (see Eq. (5)). As shown in Fig. S1, the results of the simulations significantly deviate from simulations with more mutable CpG di-nucleotides and from the empirical results. In Fig. S1(a) one can see that although the overall structure of the distribution with uniform mutation rate is similar to the empiric ones, the power-law is not as clean and possesses a rather "bumpy" shape. This is also in contrast to the result of the analytic model, which predicts a clean power-law behaviour in the asymptotic regime $s\gg 1$. Therefore, the reason for the bumps is that, in contrast to the assumption of the analytic model, not every mutation of a k-mer leads to a new, unique k-mer, but can also generate a k-mer which already exists in the genome. If one takes the non-uniform mutation rate into account in the simulations, the "bumps" almost disappear and the resulting distribution is much more similar to the empirical one and the one predicted by the analytic model [see Fig. S1(b) and Fig. 2].

So, why do CpG di-nucleotides make the power-law cleaner? The reason is as follows. Due to their high mutation rate the divergence of most Alu sequences at the CpG positions is about 36%, which is much higher than at the non-CpG positions with about 7%.[42] This makes these CpG positions similar to bps with random nucleotides. In fact, if one substitutes bps at CpG positions by random bps the resulting distribution of abundances does not change significantly, being still similar to the empirical and analytic distributions with a clean power-law. Now the question reduces to: why do a few random bps along the selfish elements smooth out the otherwise "bumpy" distribution of abundances? To understand this, let us start with a set of k-mers simulated with a uniform mutation rate resulting in the "bumpy" k-mer abundances distribution $n_k(s)$, as shown in Fig. S1(a). Consider, for simplicity, adding one random bp (say, with $1/2$ probability of C and T) to each k-mer. Then the distribution of abundances of the resulting $k+1$-mers is given by

$$\bar{n}_{k+1}(s) = 2\sum_{q=s}^{\infty}\binom{q}{s}\left(\frac{1}{2}\right)^{q} n_k(q). \tag{S9}$$

This weighted summation over $n_k(s)$ smooths the distribution, such that the resulting distribution $\bar{n}_{k+1}(s)$ has the same asymptotics, but lacks large "bumps". These qualitative results also hold if there are 4 possible nucleotides with arbitrary fractions, as long as none of the fractions is close to one. If the fraction of one nucleotide is close to one then there is no smoothing due to the summation in Eq. (S9), but merely an increase of the fraction of unique k-mers, like in summations (S7) and (S8) with a small value of p. To summarize this part we conclude, that the presence of highly mutable CpG di-nucleotides in Alu elements smooths the abundances distribution, bringing it closer to the predictions of the analytic model and the empirical results. This is why we modeled the evolution of Alu elements taking into account the presence of CpG di-nucleotides resulting in a non-uniform mutation rate along the Alu elements.

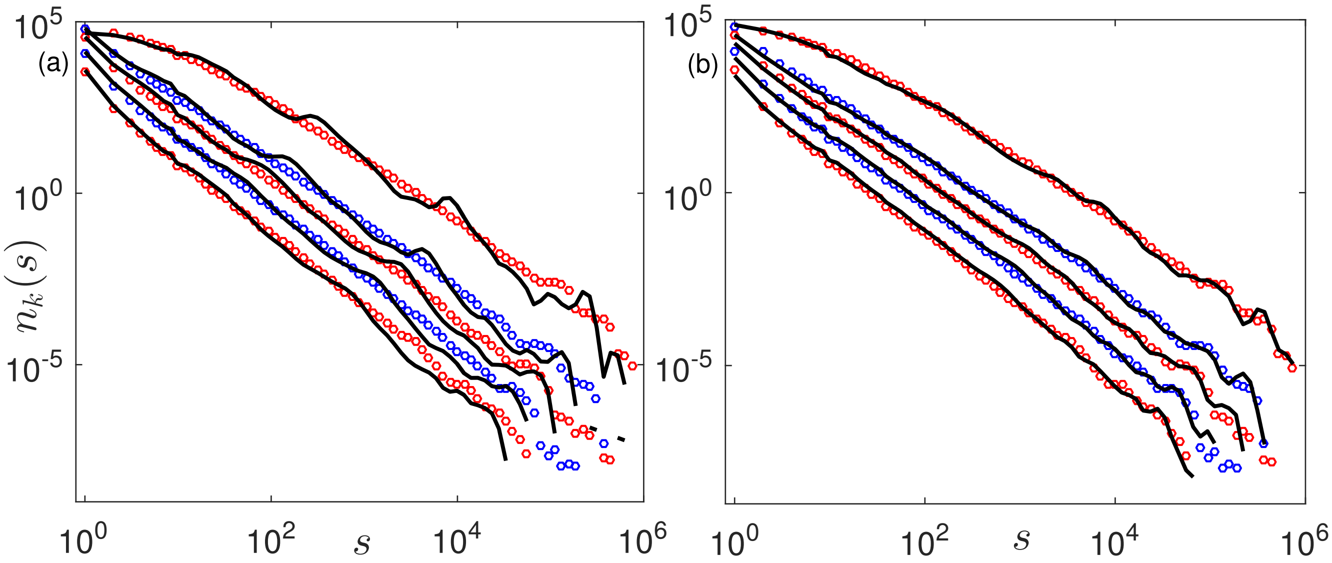

Figure S1. Distributions of abundances of k-mers for different values of k, from 10 to 30 in steps of 5, from top to bottom. Circles represent abundances of k-mers in the empirical data of Alu elements (see Methods for details). Lines represent abundances of k-mers in simulated Alu elements using the set of parameters in Eq. (9) with (a) uniform mutation rate $\mu = 1.8\mu_0$ and with (b) non-uniform mutation rate μ_0 for non-CpG nucleotides and $6\mu_0$ for CpG di-nucleotides. In fact panel (b) is merely a zoom in on Fig. 2. For visibility the values of $n_k(s)$ are normalized differently for each value of k (but in the same way for empirical and simulated data), so that the units of the vertical axis are arbitrary.

A Reproducibility of the simulations

To assess the robustness of the simulations due to their intrinsic stochasticity we performed the same simulations as in Fig. 2, but with a different random seed. The results are presented in Fig. S2. One can see that the two simulations significantly disagree only in the regions where the simulations also disagree with the empirical data (see Fig. 2).

B Other species and repeat families

The theory presented in the paper is very general and is expected to capture evolution of selfish elements in other species. To verify this we calculated distribution of k-mers for different repeat families in different species. First we compare the Alu family (again, excluding AluY) in humans and chimps. As is shown in Fig. S3, the distribution of k-mer abundances does not differ much between the two species, such that our theory can be applied also to the chimp's genome with essentially the same parameters.

For other tested species and repeat families the predictions of the theory are reaffirmed: the k-mers abundaces distributions possess a power-law tail, its power-law exponent is above 2 and grows with k, following Eq. (3). In Figs. S4,S5,S6,S7 one can see several examples of repeat families in differing species.

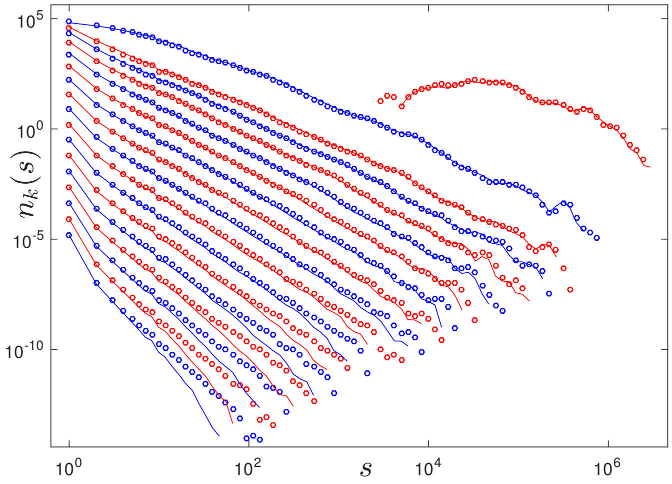

Figure S2. Distributions of abundances of k-mers, $n_k(s)$, for different values of k, from 5 to 90 in steps of 5, from top to bottom. Circles and lines represent $n_k(s)$ in simulated Alu elements using the set of parameters in Eq. (9) in Methods (as in Fig. 2). The only difference between the circles and lines is a different random seed. For visibility the values of $n_k(s)$ are normalized differently for each value of k (but in the same way for both random seeds), so that the units of the vertical axis are arbitrary.

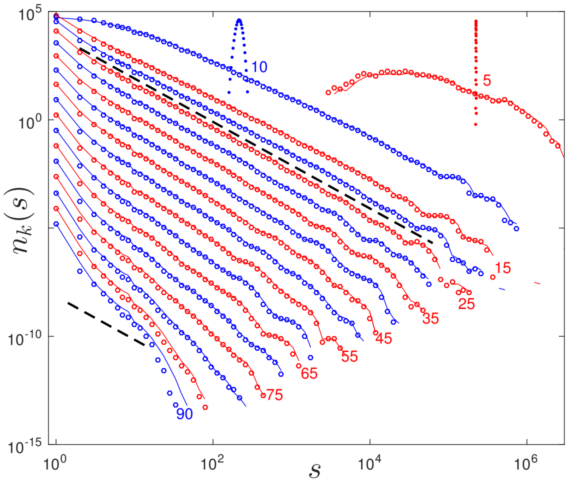

Figure S3. Distributions of abundances of k-mers, $n_k(s)$, for different values of k, from 5 to 90 in steps of 5, from top to bottom (see numbers in the figure) for huamn's and chimp's genomes. Circles (lines) represent $n_k(s)$ in the empirical data for the Alu family of repeats in the human (chimp) genome (see Methods). Dots represent $n_k(s)$ in a random sequence, of the same length as the empirical one for $k = 5$ (red) and $k = 10$ (blue). The dashed lines represent the power-law decay $n_s \sim s^{-\alpha}$ with $\alpha = 2$. For visibility the values of $n_k(s)$ are normalized differently for each value of k (but in the same way for the human and the chimp data), so that the units of the vertical axis are arbitrary.

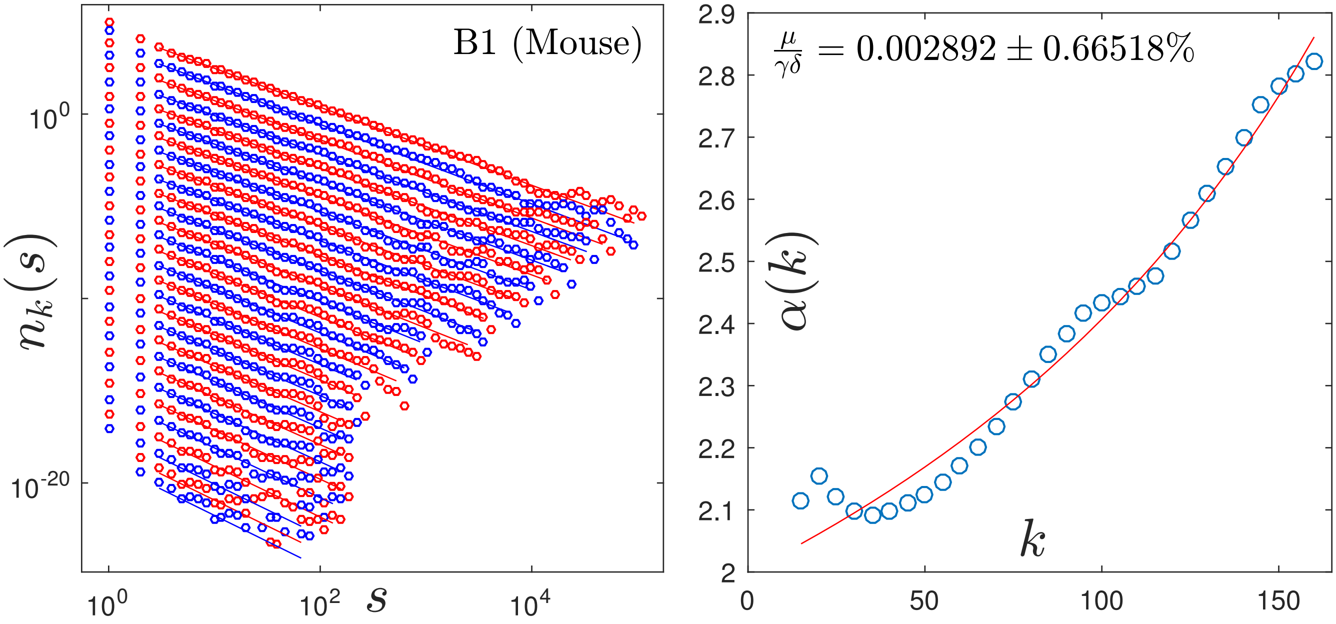

Figure S4. B1 repeat family in the M. musculus's genome. (a) Distributions of abundances of k-mers, $n_k(s)$, for different values of k, from 5 to 160 in steps of 5, from top to bottom. Circles represent $n_k(s)$ in the empirical data. Lines represent power-law fit using the Hill estimator (see methods). For visibility the values of $n_k(s)$ are normalized differently for each value of k, so that the units of the vertical axis are arbitrary. (b) Circles represent the Hill estimator of the power-law exponent α from the empirical data as a function of k. The line is the numerical fit of the data points using Eq. (3) in the $35 \leq s \leq 160$ range. The resulting estimator of $\frac{\mu}{\gamma\delta}$ is presented in the upper-left corner. For details of the estimators and the fits see Methods.

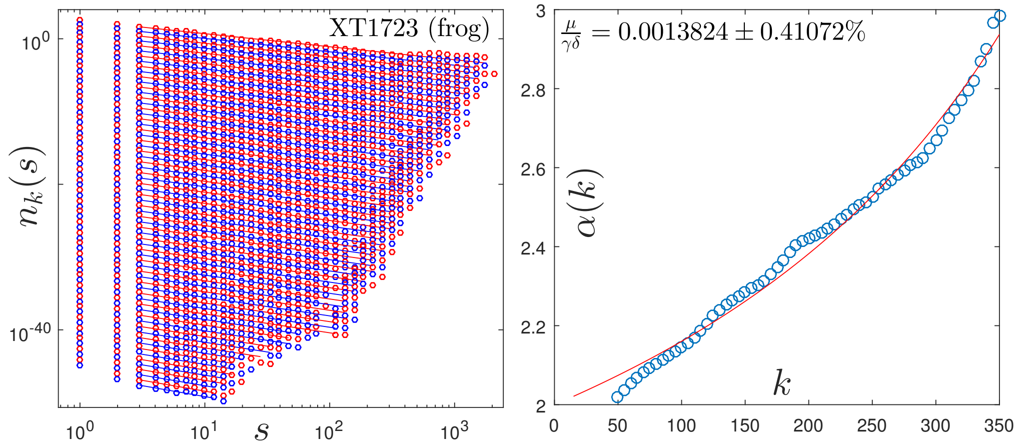

Figure S5. XT1723 repeat family in the X. tropicalis's genome. (a) Distributions of abundances of k-mers, $n_k(s)$, for different values of k, from 15 to 350 in steps of 5, from top to bottom. Circles represent $n_k(s)$ in the empirical data. Lines represent power-law fit using the Hill estimator (see methods). For visibility the values of $n_k(s)$ are normalized differently for each value of k, so that the units of the vertical axis are arbitrary. (b) Circles represent the Hill estimator of the power-law exponent α from the empirical data as a function of k. The line is the numerical fit of the data points using Eq. (3) in the $60 \leq s \leq 350$ range. The resulting estimator of $\frac{\mu}{\gamma\delta}$ is presented in the upper-left corner. For details of the estimators and the fits see Methods.

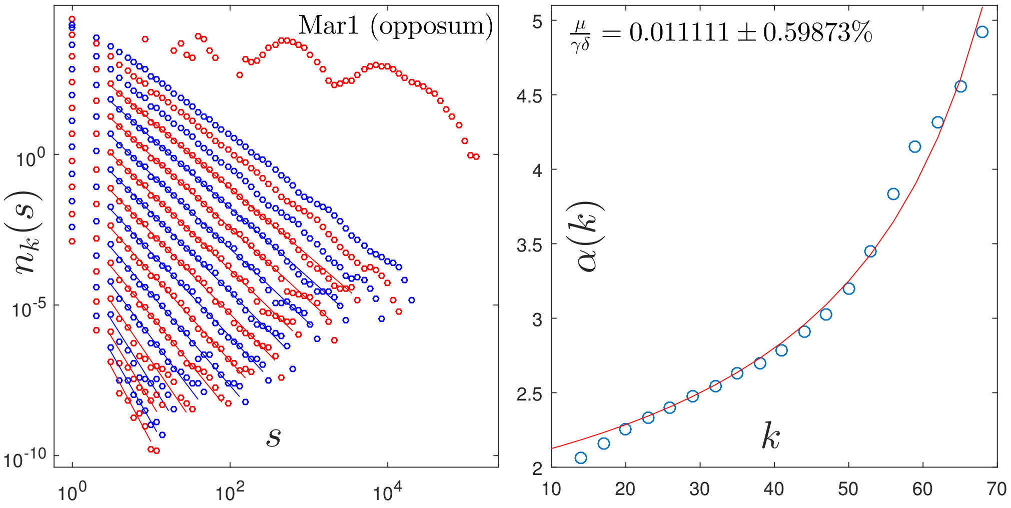

Figure S6. Mar1 repeat family in the opposum's genome. (a) Distributions of abundances of k-mers, $n_k(s)$, for different values of k, from 5 to 68 in steps of 3, from top to bottom. Circles represent $n_k(s)$ in the empirical data. Lines represent power-law fit using the Hill estimator (see methods). For visibility the values of $n_k(s)$ are normalized differently for each value of k, so that the units of the vertical axis are arbitrary. (b) Circles represent the Hill estimator of the power-law exponent α from the empirical data as a function of k. The line is the numerical fit of the data points using Eq. (3) in the $35 \leq s \leq 68$ range. The resulting estimator of $\frac{\mu}{\gamma\delta}$ is presented in the upper-left corner. For details of the estimators and the fits see Methods.

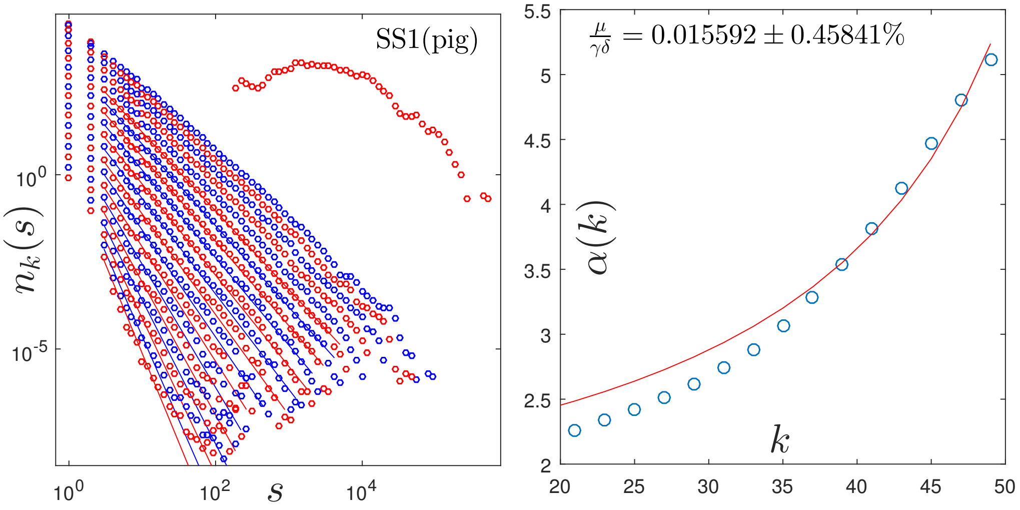

Figure S7. SS-1 repeat family in the pig's genome. (a) Distributions of abundances of k-mers, $n_k(s)$, for different values of k, from 5 to 49 in steps of 2, from top to bottom. Circles represent $n_k(s)$ in the empirical data. Lines represent power-law fit using the Hill estimator (see methods). For visibility the values of $n_k(s)$ are normalized differently for each value of k, so that the units of the vertical axis are arbitrary. (b) Circles represent the Hill estimator of the power-law exponent α from the empirical data as a function of k. The line is the numerical fit of the data points using Eq. (3) in the $35 \leq s \leq 49$ range. The resulting estimator of $\frac{\mu}{\gamma\delta}$ is presented in the upper-left corner. For details of the estimators and the fits see Methods.
